# Extracellular Production of a Novel Endo-β-Agarase AgaA from *Pseudomonas vesicularis* MA103 that Cleaves Agarose into Neoagarotetraose and Neoagarohexaose

**DOI:** 10.3390/ijms16035590

**Published:** 2015-03-11

**Authors:** Pang-Hung Hsu, Chien-Han Wei, Wen-Jung Lu, Fen Shen, Chorng-Liang Pan, Hong-Ting Victor Lin

**Affiliations:** 1 Department of Bioscience and Biotechnology, National Taiwan Ocean University, Keelung 202, Taiwan; E-Mails: phsu@mail.ntou.edu.tw (P.-H.H.); leo3220@livemail.tw (F.S.); 2 Department of Food Science, National Taiwan Ocean University, Keelung 202, Taiwan; E-Mails: hank199021@hotmail.com (C.-H.W.); miss350100@yahoo.com.tw (W.-J.L.); 3 Center of Excellence for the Oceans, National Taiwan Ocean University, Keelung 202, Taiwan

**Keywords:** agar, agarase, neoagaro-oligosaccharides, *Pseudomonas vesicularis*, osmotic shock, extracellular

## Abstract

The gene *agaA*, of the isolated marine bacterium *Pseudomonas vesicularis* MA103, comprised 2958-bp nucleotides encoding a putative agarase AgaA of 985 amino acids, which was predicted to contain a signal peptide of 29 amino acids in the *N*-terminus, a catalytic domain of glycoside hydrolase 16 (GH16) family, a bacterial immunoglobulin group 2 (Big 2), and three carbohydrate binding modules 6 (CBM 6). The gene *agaA* was cloned and overexpressed in *Escherichia coli*, and the optimum temperatures for AgaA overexpression were 16, 20 and 24 °C. The *agaA* was cloned without its signal peptide for cytosolic production overexpression, whereas it was cloned with the heterologous signal peptide PelB and its endogenous signal peptide for periplasmic and extracellular productions, respectively. Extracellular and periplasmic rAgaA showed greater activity than that of cytosolic rAgaA, indicating that membrane translocation of AgaA may encourage proper protein folding. Time-course hydrolysis of agarose by rAgaA was accomplished and the products were analyzed using thin layer chromatography and matrix-assisted laser desorption inoization-time of flight mass spectrometry, indicating that AgaA from *P. vesicularis* was an endo-type β-1,4 agarase that cleaved agarose into neoagarotetraose and neoagarohexaose as the final products.

## 1. Introduction

Natural sources from marine environment have attracted great attention worldwide. Agar comprising two different polysaccharides, *i.e.*, agarose and agaropectin, was found in the cell walls of red algae [1]. Agarose is a neutral linear polysaccharide composed of a repeating unit of agarobiose, a disaccharide made up of 4-*O*-linked 3,6-anhydro-α-l-galactopyranose (A-unit) and 3-*O*-linked β-d-galactopyranose (G-unit) [1]. Agarose can be hydrolyzed by α-agarase and by β-agarase; the former cleaves the α-1,3 linkage of agarose to generate agaro-oligosaccharides, and the latter cleaves the β-1,4 linkage to generate neoagaro-oligosaccharides [2]. To date, neoagaro-oligosaccharides have been found to exhibit various biological and physiological functions, such as moisturizing effect on skin [3], whitening effect on melanoma cells [4], anti-inflammation [5], inhibition of bacterial growth, and decrease of starch degradation [6]. Apart from the use of agarases in the production of oligosaccharides, they are brilliant biological tools for DNA recovery from agarose gel and for the preparation of protoplasts from red algae [7].

Several agarases have been identified and characterized from various genera, including the archaea *Halococcus* [8], Gram-positive bacteria, such as *Bacillus* [9], *Paenibacillus* [10], *Rhodococcus* [11] and *Streptomyces* [12], and Gram-negative bacteria, such as *Acinetobacter* [13], *Agarivorans* [14,15], *Alteromonas* [16], *Catenovulum* [17], *Flammeovirga* [18], *Janthinobacterium* [19], *Microbulbifer* [20], *Pseudomonas* [21], *Pseudoalteromonas* [22], *Saccharophagus* [23], *Thalassomonas* [24], *Vibrio* [25], and *Zobellia* [26]. So far, in published reports and CAZy database [27], the identified β-agarases have been more abundant than α-agarases. Based on their protein sequence similarities, β-agarases can be generally categorized into four glycoside hydrolase (GH) families (*i.e*., GH16, GH50, GH86, and GH118 [28]), and their major smallest end products were reported to be neoagarotetraose (NA4) [29], neoagarobiose (NA2) [4], neoagarohexaose (NA6) [20], and neoagaro-octaose (NA8) [30], respectively. The GH16 family, the largest family among the four families, comprises more than 3000 members that are functionally heterogeneous, such as endoglucanase, endo-galactosidase, β-agarases, β-galactanase, κ-carrageenase, β-porphyranase, and xyloglucanase [27].

*Pseudomonas vesicularis* MA103 was previously isolated from the seawater off the coast of Keelung in Taiwan, and its genome was sequenced [31]. It was revealed that a gene of 2958 bp encoding a putative agarase had a significant homology to the β-agarase of GH16 family. AgaA from *Vibrio* spp. PO-303, a homologous agarase of the AgaA from *P. vesicularis* MA103, was successfully cloned and overexpressed without its endogenous signal peptide in *Escherichia coli*, and cytosolic *Vibrio* AgaA was collected after cell disruption [29]. The optimum pH and temperature conditions for *Vibrio* AgaA activity were characterized, and the primary kinetic parameters were determined. However, the possible biological importance of the endogenous signal peptide and translocation to the periplasm remained elusive. Moreover, the end neoagaro-oligosaccharides products of the *Vibrio* AgaA have not been precisely determined. In this study, novel *agaA* gene from *P. vesicularis* was cloned and overexpressed in *E. coli* without or with a signal peptide (heterologous and endogenous), and the overexpressed AgaAs from different clones were collected in the cytoplasm, periplasm, and extracellular space. The agarase activity of recombinant AgaAs was determined and the biological importance of the protein translocation and end products of AgaA activity was described.

## 2. Results and Discussion

### 2.1. Domain Prediction of AgaA from P. vesicularis MA103

**Figure 1:**
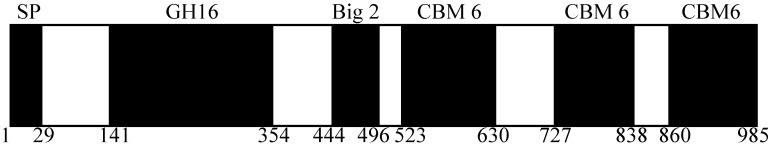
Predicted domain structure of AgaA from *P. vesicularis* MA103. SP, signal peptide; GH16, glycoside hydrolase module of family 16; Big-2, bacterial immunoglobulin group 2 domain; CBM 6, carbohydrate-binding module 6. The amino acid residues of each domain are indicated below the figure.

The gene *agaA* comprised 2958 bp nucleotides encoding an agarase of 985 amino acids, and a predicted ribosome-binding site (5'-AGGACG-3') was found at 7 bp upstream of the ATG start codon. BLASTp [32] analysis indicated that the degree of amino acid sequence identity between AgaA from *P. vesicularis* MA103 and that from *Vibrio* spp. PO-303 [29] was 99%, suggesting that these two agarases are homologous proteins. According to the SignalP server [33], AgaA from *P. vesicularis* MA103 was predicted to have a signal peptide of 29 amino acids at its *N*-terminus. As shown in Figure 1, the protein sequence of AgaA from *P. vesicularis* MA103 was analyzed using Pfam, indicating that the protein comprised the catalytic domain of GH16 family (from 141 to 354 amino acid residues) and a bacterial immunoglobulin group 2 (Big 2) domain (from 444 to 496 amino acid residues), followed by three carbohydrate binding modules 6 (CBM 6). The CBM 6 located on the *C*-terminus of the protein comprised a complete CBM 6 sequence, while the other two were truncated. The CBM 6 modules contain approximately 120 residues and possess cellulose-binding function, which has been previously demonstrated with amorphous cellulose and β-1,4-xylan [34]. AgaA from *P. vesicularis* displayed the catalytic residues Glu^232^, Asp^234^, and Glu^237^ within the catalytic sequence motif eldvyeqsgrrs (from 232 to 243 amino acid residues), which was highly homologous to the conserved sequences E[ILV]D[IVAF]X(0,1)E of the agarases that belonged to the GH16 family [35,36].

### 2.2. Cloning

**Figure 2:**
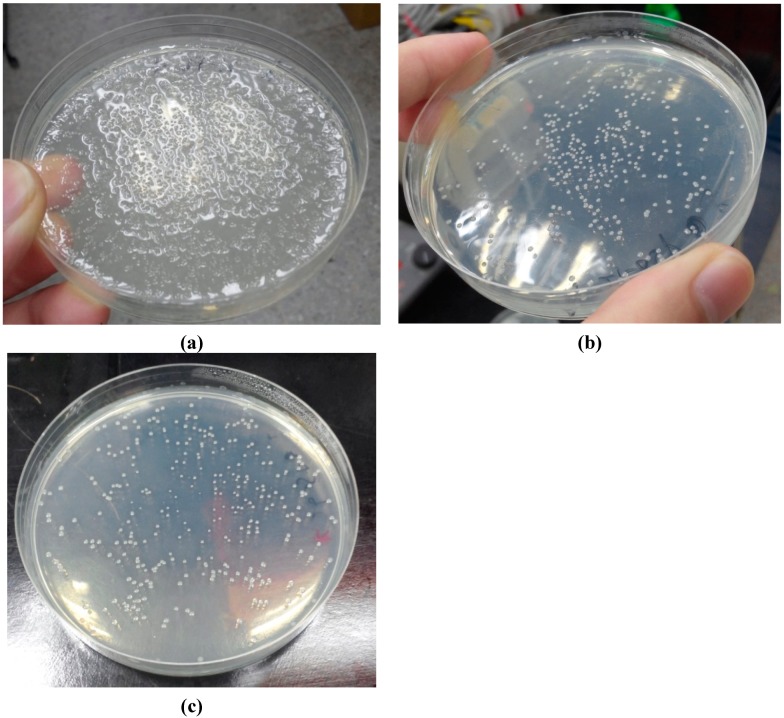
The agar plates grown with *E. coli* harboring (**a**) pET-AgaA-FL (**b**) pET-AgaA-ΔSP and (**c**) pET-AgaA-PelB. *E. coli* cells harboring the three different plasmids, individually, were plated out onto Luria-Bertani (LB) agar plates and incubated at 37 °C for 12 h.

The secretion of heterologous extracellular proteins in *E. coli* is in high demand for enzyme production because it has the advantage of minimizing toxicity problems to the host during protein overexpression, avoiding the formation of inclusion bodies, and of simplifying further purification [37,38]. In a previous report [29], truncated AgaA from *Vibrio* spp. PO-303 was successfully cloned without its endogenous signal peptide, overexpressed in *E. coli*, and recovered in the cytosol after cell disruption. Considering that AgaA from *P. vesicularis* is an extracellular enzyme, we constructed three plasmids encoding AgaA to determine the possible biological significance of a signal peptide to the activity of AgaA: pET-AgaA-FL (full length), pET-AgaA-ΔSP (deletion of the 29 amino acid residues at the *N*-terminus), and pET-AgaA-PelB (truncated AgaA fused to a heterologous signal peptide PelB at the *N*-terminus). The *E. coli* transformants harboring pET-AgaA-FL caused degradation of the agar plate (Figure 2), indicating that AgaA-FL was overexpressed and it might be secreted into the extracellular spaces, which were not observed in the *E. coli* cells overexpressing AgaA-ΔSP and Aga-SP.

### 2.3. Effect of Temperature on AgaA Overexpression

**Figure 3:**
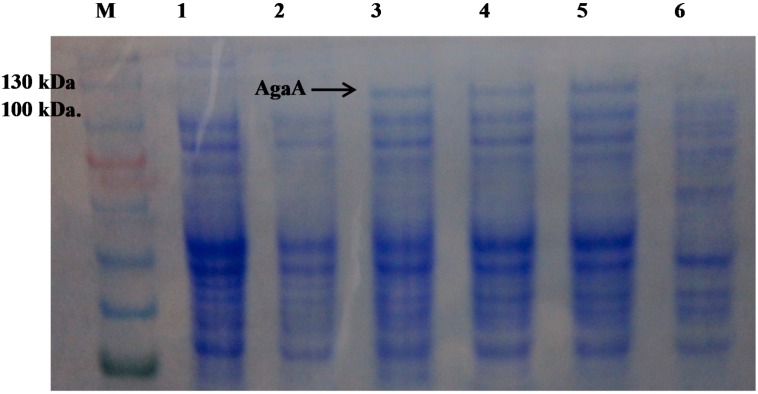
The sodium dodecyl sulfate polyacrylamide gel electrophoresis (SDS-PAGE) of overexpressed pET-AgaA-ΔSP at various temperatures. Lane **M**, protein marker; Lane **1**, C43 (DE3) harboring an empty plasmid; Lane **2**, uninduced C43 (DE3)/pET-AgaA-ΔSP; Lane **3**–**6**, C43 (DE3)/pET-AgaA-ΔSP induced with 0.2 mM IPTG at different temperatures, respectively (16, 20, 24 and 37 °C). The *E. coli* cells harboring pET-AgaA-ΔSP were induced by 2 mM IPTG at various temperatures for 12 or 6 h (37 °C), and the overexpression of AgaA-ΔSP were analyzed by using SDS-PAGE.

The effect of temperature on AgaA overexpression in *E. coli* was studied by inducing *E. coli* harboring pET-AgaA-ΔSP at 16, 20, 24 and 37 °C. The cells were harvested and analyzed using SDS-PAGE. As shown in Figure 3, no overexpression of AgaA was observed in *E. coli* harboring empty plasmid and uninduced *E. coli* harboring pET-AgaA-ΔSP (Lanes 1 and 2). Overexpression of putative AgaA-ΔSP was observed at the induction temperatures of 16, 20 and 24 °C (Lanes 3–5), but it was not observed at that of 37 °C (Lane 6). The estimated molecular weight of overexpressed AgaA-ΔSP was 103.4 kDa, and it was accordingly located between the 100 and 130 kDa protein marker bands on the SDS-PAGE. Because the putative AgaA-ΔSP overexpression at 16, 20, and 24 °C showed no obvious differences in the overexpression level, 24 °C was chosen for AgaA overexpression for the rest of the study.

### 2.4. Effect of the N-Terminal Signal Peptide on the Overexpression of Functional AgaA

**Figure 4:**
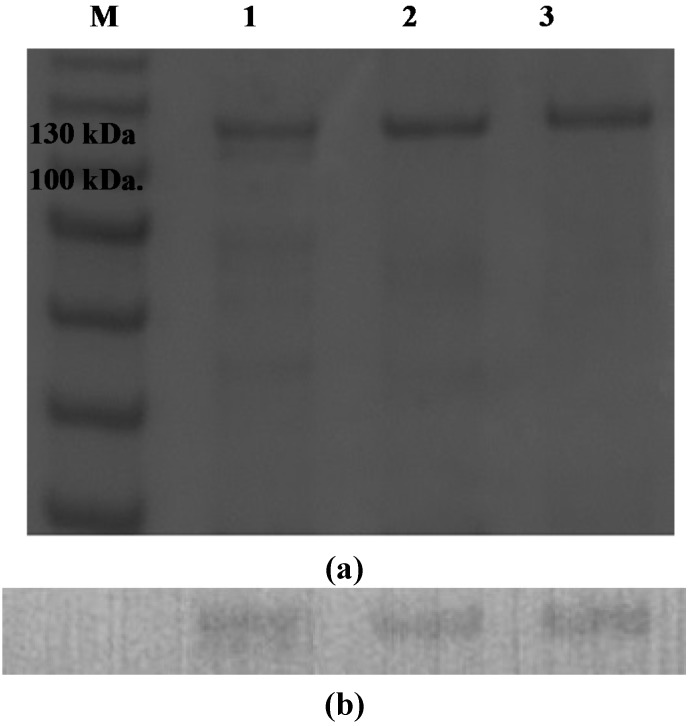
The (**a**) SDS-PAGE and (**b**) Western blot of AgaA. Lane **M**, protein marker; Lane **1**, AgaA-FL; Lane **2**, AgaA-ΔSP; Lane **3**, AgaA-PelB.

As shown in Figure 4, the purified recombinant agarases (rAgaAs) AgaA-FL (Lane 1), AgaA-ΔSP (Lane 2), and AgaA-PelB (Lane 3) were visualized with SDS-PAGE and identified using Western blot. The three rAgaAs were all located between the 100 and 130 kDa marker bands, indicating that they have similar protein sizes. Overexpressed AgaA-FL and AgaA-PelB were recovered from the culture medium and *E. coli* periplasm, respectively, whereas AgaA-ΔSP that was not found in the periplasm or culture medium was collected from the cytoplasm. The agarase activities of the culture medium, whole cells and the fraction of the whole cells extracts (*E. coli* harboring pET-AgaA-FL) were compared, and the activity for the culture medium was significantly higher than those ones for the whole cells and the fraction of whole cell extracts, suggesting that AgaA-FL was secreted to the culture broth. Our data suggested that the endogenous peptide of AgaA from *P. vesicularis* led AgaA-FL into the culture medium, the PelB leader sequence led AgaA-PelB into the cell periplasm, and AgaA-ΔSP failed to translocate across the inner membrane because of missing signal peptide. The specific agarase activity of the purified AgaA-FL, AgaA-ΔSP, and AgaA-PelB were determined to be 425.6 ± 4.8, 37.7 ± 0.1, and 132.6 ± 2.9 units/mg, respectively (Table 1). It was evident that AgaA-FL and AgaA-PelB, which translocated across the inner membrane during overexpression, display a better agarase activity than that displayed by the cytosolic AgaA-ΔSP does. In most bacteria, Sec-dependent [39] and twin-arginine translocation (Tat) pathway [40] are the two predominant routes for protein export. Proteins targeted to the Sec and Tat pathway normally possess a tripartite *N*-terminal signal peptide comprising a hydrophobic core region (h-region) flanked by a positively charged *N*-terminal and polar *C*-terminal region [39,40]. The signal peptide targeting the Tat pathway has a conserved twin-arginine motif in the *N*-terminal region that was not found in the signal sequence of the full-length AgaA from *P. vesicularis*, demonstrating that AgaA translocation may belong to the sec-dependent pathway by which unfolded proteins are translocated. Our activity data suggested that the translocation of AgaA across the inner membrane was important for the agarase activity, possibly because of the induction of protein folding during protein translocation. A review article on agarases of various organisms has also reported that periplasmic and extracellular recombinant GH16 AgaA has greater average agarase specific activity (160–517 U/mg) than that of the cytosolic recombinant AgaA (16.4–32.3 U/mg) [2].

**Table 1:** Agarase activity of recombinant AgaA from *P. vesicularis*.

Protein Source *	Total Protein (mg)	Total Activity (units)	Specific Activity (units/mg)
AgaA-FL	12.2	5192.3	425.6
AgaA-∆SP	18.3	689.9	37.7
AgaA-PelB	6.2	816.8	132.6

* The proteins were purified from one liter of cells cultures.

### 2.5. AgaA from P. vesicularis Is an Endo-Type β-Agarase Yielding Neoagarotetraose and Neoagarohexaose Products

**Figure 5:**
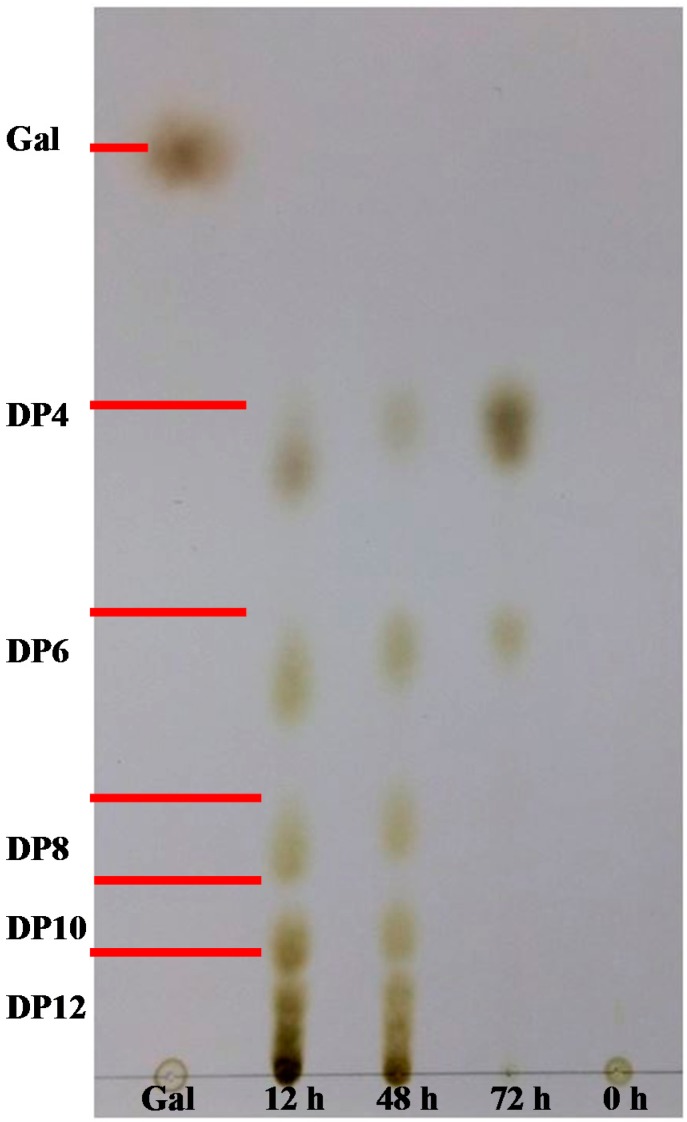
TLC chromatogram of the products at different time points of agarose hydrolysis by recombinant AgaA-FL. The sample was prepared by incubating recombinant AgaA with 0.3% (*w*/*v*) agarose at 40 °C for 72 h.

**Figure 6:**
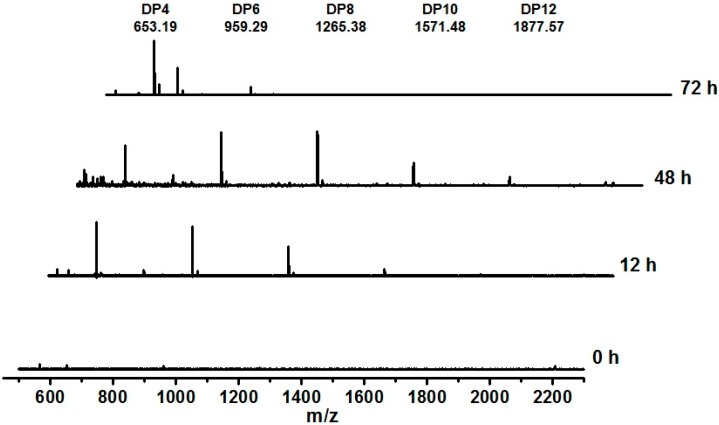
MALDI-TOF mass spectrum of the products at different time points of agarose hydrolysis by recombinant AgaA-FL. The sample was prepared by incubating recombinant AgaA with 0.3% (*w*/*v*) agarose at 40 °C for 72 h.

Endo-type agarase is known to randomly degrade agarose and thus rapidly lower the viscosity of agarose solutions, whereas exo-type agarase tends to produce single major products and gradually decrease the viscosity of agarose solutions [23,41]. Most GH16 agarases are reported to be endo-type agarases [2]. To ascertain whether AgaA from *P. vesicularis* was an endo-type or exo-type agarase as well as to determine its end hydrolysis products, a time-course hydrolysis from 0 to 72 h was performed using purified AgaA. As shown in Figure 5, the thin layer chromatography (TLC) data obtained from this study indicated that agarose hydrolysis by AgaA for 12 and 48 h yielded products with various degrees of polymerization (DP), including DP4 (neoagarotetraose), DP6 (neoagarohexaose), DP8, DP10, DP12, and higher oligosaccharides. The products obtained after hydrolysis for 72 h indicated that only DP4 and DP6 remained in the reaction solution, suggesting that DP4 and DP6 were the end products of the agarose hydrolysis by AgaA from *P. vesicularis*. Furthermore, we used MALDI-TOF MS to identify the products during agarose hydrolysis. As shown in Figure 6, the products at 12 and 48 h of incubation were of various DP, such as DP4, DP6, DP8, DP10, and DP12 oligosaccharides. With the increase in the incubation time, the reducing sugar increased, indicating continuous agarose hydrolysis (data now shown). The cleavage patterns and proportions of each oligosaccharide were similar up to 48 h, indicating that AgaA may have greater affinity to agarose rather than to oligosaccharides. At 48 h, the agarose solution was filtered out, and recombinant AgaAs were added again. The portions of the larger oligosaccharides (*i.e*., DP8, DP10, and DP12) gradually decreased, while those of the smaller products DP4 and DP6 remained, indicating that the end products of AgaA hydrolysis were neoagarohexaose and neoagarotetraose.

GH50 agarase Aga50D from *Saccharophagus degradans* exhibited exo-type β-agarase activity by showing constant production of neoagarobiose during time-course agarose hydrolysis [23]. YM01-3 from the marine bacterium *Catenovulum agarivorans* YM01 and AgaB from *Pseudoalteromonas* spp. CY24 were reported to be GH16 endo-type β-agarases [30,36] by showing their analyses of time-course agarose hydrolysis using TLC. YM01-3 and AgaB hydrolyzed the β-1,4-glycosidic linkages of agarose to generate neoagarodecaoses, neoagarooctaoses, neoagarohexaoses, and neoagarotetraoses, and the amount of DP6 and DP4 increased in a time-dependent manner [30,36,41]. The TLC and MS data of this study indicated that AgaA from *P. vesicularis* is an endo-type β-1,4 agarase, with neoagarohexaose and neoagaroteraose as end products.

## 3. Experimental Section

### 3.1. Isolation of Pseudomonas vesicularis MA103

The bacterial strain *P. vesicularis* MA103 was isolated from the seawater off the coast at Keelung in Taiwan, and its genome was sequenced by Mission Biotech Ltd. (Taipei, Taiwan).

### 3.2. Cloning of AgaA

The *agaA* gene was cloned from the *P. vesicularis* MA103 chromosome by using PCR method and three plamids encoding AgaA were constructed. The full length *agaA* gene amplified by using the primers 5'-CATATGTCTACACAATGGCCTAAGTCGCCA-3' and 5'-CTCGAGTTGCTTGGTTAGGGTGAATTTGTC-3', was digested with NdeI and XhoI restriction enzymes, and inserted onto pET21a vector at the NdeI-XhoI site to generate the plasmid pET-AgaA-FL (full length) for the overexpression of AgaA-FL. The truncated *agaA* gene (87 bp deletion at 5' end) was amplified using the primers 5'-CATATGATTTATACACCTTGGGAAGGCTCTTTC-3' and 5'-CTCGAGTTGCTTGGTTAGGGTGAATTTGTC-3', the PCR products were digested with NdeI and XhoI restriction enzymes and inserted into pET21a vector at the NdeI-XhoI site to generate pET-AgaA-ΔSP for the overexpression of AgaA-ΔSP (deletion of the 29 amino acids residues at the *N*-terminal end). The third constructed by amplifying the truncated *agaA* gene (87 bp deletion at 5' end) with the primers 5'-CCATGGTGATTTATACACCTTGGGAAGGC-3' and 5'-CTCGAGTTGCTTGGTTAGGGTGAATTTGTC-3', the PCR products were digested with NcoI and XhoI restriction enzymes, and inserted into pET26b vector at the NcoI-XhoI site to generate pET-AgaA-PelB for the overexpression of AgaA-PelB (truncated AgaA fused a heterologous signal peptide of pectate lyase B (PelB) from *Erwinia carotovora* into its *N*-terminus). The plasmids encoding β-agarase AgaA were transformed into *E. coli* C43 (DE3) for protein overexpression.

### 3.3. AgaA Overexpression and Purification

The optimum temperatures for the overexpression of AgaA from *P. vesicularis* MA103 was investigated a temperatures (16, 20, 24 and 37 °C) and the *E. coli* C43(DE3) harboring the plasmids encoding AgaA was overexpressed upon IPTG induction. The three AgaA plasmids, pET-AgaA-FL, pET-AgaA-ΔSP and pET-AgaA-PelB, were purified using different strategies. The overexpressed extracellular AgaA-FL was collected in the growth media. The induced cells harboring pET-AgaA-ΔSP were collected by using centrifugation and re-suspended in the Tris-HCl buffer containing 200 mM NaCl. The cells were disrupted using sonication and the supernatant containing overexpressed AgaA-ΔSP were collected after centrifugation. The induced cells harboring pET-AgaA-PelB were collected and re-suspended in the Tris-HCl buffer containing 20% sucrose and EDTA, pH 8.0 (Osmotic shock buffer 1), and the cells were pelleted again and re-suspended and incubated in the Tris-HCl buffer containing 5 mM MgSO_4_ (Osmotic shock buffer 2) for 30 min. The recombinant AgaA-PelB was released into the buffer and purified using Nickel affinity column (Hitrap chelating column, GE Healthcare, Piscataway, NJ, USA) or gel filtration (HiLoad 16/600 Superdex 200, GE Healthcare) where necessary.

### 3.4. Western Blot

AgaA proteins were first analyzed by using gel electrophoresis. The proteins were transferred to a PVDF (Polyvinylidene difluoride, GE Healthcare) membrane and probed with the primary antibody, a mouse monoclonal anti-polyhistidine antibody (GE Healthcare). The PVDF membrane was then incubated with a second antibody, goat anti-mouse immunoglobulin G AP (alkaline phosphatase) conjugate, and the proteins were identified using a colorimetric alkaline phosphatase conjugate substrate kit (Bio-Rad, Hercules, CA, USA).

### 3.5. Enzyme Activity Assay

The agarase activity of AgaA was measured according to Miller [42] with some modifications. Fifty microliters of AgaA was incubated with 450 μL agarose (0.3%, *w*/*v*) in 0.1 M phosphate buffer (pH 7) at 40 °C for 10 min. The reaction mixture was added to 500 μL 3,5-dinitrosalicylic acid solution (DNS) solution and incubated at 100 °C for 5 min to stop reaction and for color development, which was measured at OD_546_. The amount of formed reducing sugar was determined by DNS using d-galactose as standard. Enzyme activity (Unit) was defined as the amount of enzyme required to liberate 1 μmol d-galactose per minute, and the specific activity was defined as the activity unit per milligram of enzyme (units/mg).

### 3.6. Time Course Hydrolysis of Agarose

Prepared 0.3% (*w*/*v*) agarose were added with recombinant AgaA from *P. vesicularis* and incubated at 40 °C. The hydrolysis products were taken at 0, 3, 6, 12, 24, 48, 60, and 72 h. The samples were filtered out to remove any solids to be ready for analysis.

### 3.7. Thin-Layer Chromatography (TLC)

Samples from the hydrolysis reaction were taken at different time points and applied onto Silica Gel 60 TLC plates (Merck, Darmstadt, Germany). Plates were developed with an *n*-butanol–acetic acid–water solution (with a mixing ratio of 2:1:1 in volume). The hydrolyzed products were detected by 10% (*v*/*v*) H_2_SO_4_ in ethanol, followed by heating at 110 °C for 15 min.

### 3.8. Matrix-Assisted Laser Desorption/Ionization Time-of-Flight Mass Spectrometry (MALDI-TOF MS)

The hydrolysis products of agarose by recombinant AgaA-FL were collected at the reaction time 0, 12, 48 and 72 h. The molecular weights of the hydrolysis products were examined by MALDI-TOF mass spectrometer (Autoflex II, Bruker Daltonics Inc., Billerica, MA, USA). 2,5-Dihydroxybenzoic acid (DHB) was used as the matrix for hydrolysis products. The MS spectra were internally calibrated by tryptic bovine serum albumin peptides, which resulted in mass errors of less than 50 ppm. All MS data were processed by Bruker Data Analysis (ver. 4.2, Bruker Daltonics Inc.).

### 3.9. Statistical Analysis

Data were statistically analyzed using SPSS Version 12.0 (SPSS Inc., Chicago, IL, USA). One-way analysis of variance (ANOVA) was used to determine the statistical differences between the sample means, with the level of significance set at *p <* 0.05. Multiple comparisons of the means were conducted using the Tukey test. All data are expressed as mean ± SD.

## 4. Conclusions

Various agarases have been identified and isolated for the production of neoagarooligosaccharides that have been found to exhibit various biological and physiological functions. AgaA from *P. vesicularis*, a new endo-type β-1,4 agarase, was cloned and overexpressed in *E. coli*, and it was shown to cleave agarose into neoagarohexaose and neoagaroteraose as the end products. The extracellular production of recombinant *P.* *vesicularis* AgaA was found to possess agarase activity higher than that of cytosolic AgaA. This result is of particular interest because the secretion of extracellular proteins for enzyme production can avoid the costs for enzyme recovery from disrupted cells and further purification. Our study identified and characterized a new β-agarase from *P. vesicularis* and provided a practical strategy for the extracellular production of the enzyme.
